# Human plasma-like medium facilitates metabolic tracing and enables upregulation of immune signaling pathways in glioblastoma explants

**DOI:** 10.1101/2023.05.29.542774

**Published:** 2023-05-31

**Authors:** Mohamad El Shami, Milan R Savani, Lauren C Gattie, Bailey Smith, William H Hicks, Jeremy N Rich, Timothy E Richardson, Samuel K McBrayer, Kalil G Abdullah

**Affiliations:** 1 Department of Neurosurgery, University of Pittsburgh School of Medicine, 200 Lothrop St, Pittsburgh, PA, 15213, USA.; 2 Hillman Comprehensive Cancer Center, University of Pittsburgh Medical Center, 5115 Centre Ave, Pittsburgh, PA, 15232, USA.; 3 Children’s Medical Center Research Institute, University of Texas Southwestern Medical Center, 6000 Harry Hines Blvd, Dallas, TX, 75235, USA.; 4 Simmons Comprehensive Cancer Center, University of Texas Southwestern Medical Center, Dallas, TX, 75390, USA.; 5 Department of Pathology, Molecular, and Cell-Based Medicine, Icahn School of Medicine at Mount Sinai, New York, NY, 10029, USA.

**Keywords:** Glioma, organoid, metabolism, stable isotope tracing

## Abstract

**Purpose:**

Metabolism within the tumor microenvironment (TME) represents an increasing area of interest to understand glioma initiation and progression. Stable isotope tracing is a technique critical to the study of tumor metabolism. Cell culture models of this disease are not routinely cultured under physiologically relevant nutrient conditions and do not retain cellular heterogeneity present in the parental TME. Moreover, in vivo, stable isotope tracing in intracranial glioma xenografts, the gold standard for metabolic investigation, is time consuming and technically challenging. To provide insights into glioma metabolism in the presence of an intact TME, we performed stable isotope tracing analysis of patient-derived, heterocellular Surgically eXplanted Organoid (SXO) glioma models in human plasma-like medium (HPLM).

**Methods:**

Glioma SXOs were established and cultured in conventional media or transitioned to HPLM. We evaluated SXO cytoarchitecture and histology, then performed spatial transcriptomic profiling to identify cellular populations and differential gene expression patterns. We performed stable isotope tracing with ^15^N_2_-glutamine to evaluate intracellular metabolite labeling patterns.

**Results:**

Glioma SXOs cultured in HPLM retain cytoarchitecture and cellular constituents. Immune cells in HPLM-cultured SXOs demonstrated increased transcription of immune-related signatures, including innate immune, adaptive immune, and cytokine signaling programs. ^15^N isotope enrichment from glutamine was observed in metabolites from diverse pathways, and labeling patterns were stable over time.

**Conclusion:**

To enable ex vivo, tractable investigations of whole tumor metabolism, we developed an approach to conduct stable isotope tracing in glioma SXOs cultured under physiologically relevant nutrient conditions. Under these conditions, SXOs maintained viability, composition, and metabolic activity while exhibiting increased immune-related transcriptional programs.

## Introduction

A hallmark of cancer biology is metabolic reprogramming to provide energy and critical components for cell growth [1–3]. There has recently been a resurgence of interest in the metabolism of glioma cells and a recognition of the importance of metabolic adaptations that enable cells to grow and proliferate. Metabolic studies of glioma can provide valuable insight into tumor metabolism and unravel new treatment targets [2]. Intracellular metabolic networks are flexible and sensitive to the concentrations of environmental metabolites [4–6]. The current gold standard for investigating tumor metabolism dynamics in the presence of an intact TME entails in vivo stable isotope tracing in mouse models of cancer [7]. These experiments, however, are technically challenging, costly, and time-consuming. More tractable methods for interrogating cancer cell metabolism, such as cell culture models, fail to capture vital contributors to tumor biology, including heterocellular interactions of the tumor microenvironment (TME) and physiologic nutrient levels.

Surgically eXplanted Organoids (SXOs) have emerged as an attractive model to study glioma metabolism. SXOs are efficiently created without mechanical or enzymatic single-cell dissociation of the resected tumor tissue, allowing for maintenance of local cytoarchitecture and native cell-cell interactions [8]. These models recapitulate parental tumor features such as cellular heterogeneity, cell-cell and cell-stroma interactions, gene expression and mutational profiles, and treatment response [8–10]. Glioma SXOs are traditionally cultured in the nutrient-rich, non-physiologic medium glioma organoid complete (GOC). SXO metabolism has not been interrogated in settings that more closely mirror the parental tumor metabolic milieu. Recently, several studies have described the use of media designed to better reflect the physiologic environment in various cancer models [1, 6, 11–13]. Among these is human plasma-like medium (HPLM), which contains physiologically relevant concentrations of typical media components such as glucose, amino acids, and salt ions, as well as metabolites absent from commonly used culture media.

**Fig. 1:**
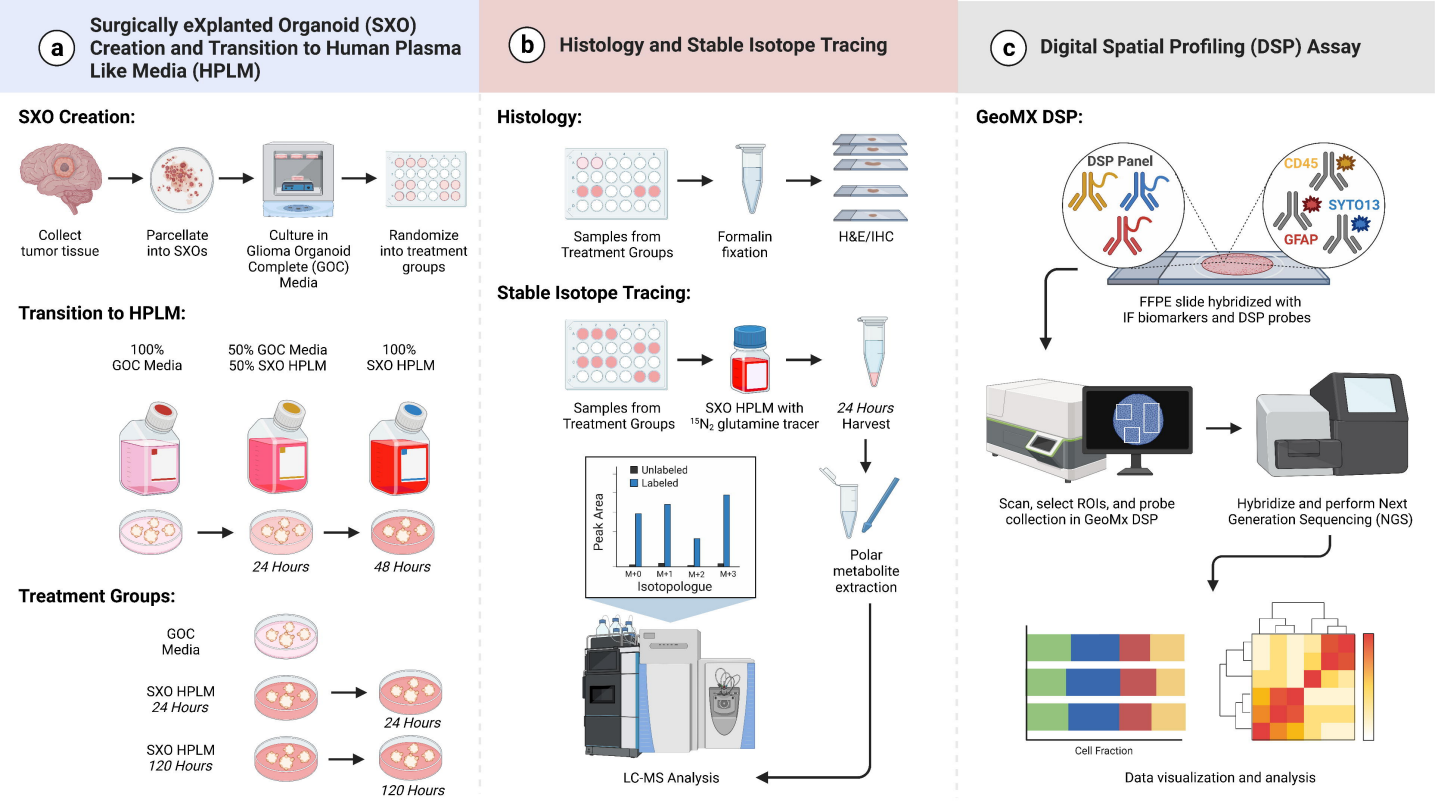
Overview of experimental scheme Glioblastoma Surgically eXplanted Organoid (SXO) creation, SXO transition to SXO Human Plasma Like Medium (HPLM), and analysis using histology, spatial transcriptomics, and stable isotope tracing. **(a)** SXO creation and transition to HPLM. Human brain tumor tissue was collected directly from the operating room, manually parcellated, and established in culture. SXOs were cultured in Glioma Organoid Complete Medium (GOC) for 4 weeks, then randomized to culture in GOC or HPLM. SXOs in HPLM were first transferred into adaptation media containing 50% GOC and 50% SXO HPLM for a 24-hour adaptation period, then into 100% SXO HPLM and cultured for either an additional 24 or 120 hours. **(b)** Histology and Stable Isotope Tracing. *Histology:* SXOs were fixed using 10% neutral buffered formalin for one hour, then washed four times with 70% ethanol and transferred to cryotubes containing 70% ethanol. SXO were stained for hematoxylin and eosin (H&E), Ki67, and Sox2. *Stable Isotope Tracing:* SXOs cultured in SXO HPLM for 24 or 120 hours were transferred into SXO HPLM with glutamine exchanged for equimolar ^15^N_2_ isotopically labeled glutamine. After 24 hours of exposure to labeled SXO HPLM, polar metabolites were harvested by manual agitation in 80% acetonitrile. Extracted metabolites were immediately subjected to liquid chromatography-mass spectrometry (LC-MS). **(c)** Digital Spatial Profiling (DSP) Assay. Formalin-fixed paraffin-embedded tissue slides were deparaffinized and stained with fluorescent antibodies and antibodies coupled to DNA-barcoded oligos (SYTO13 to identify DNA, GFAP to identify tumor cells, and CD45 to identify immune cells). Fluorescent section images were collected, then spatially resolved regions of interest (ROIs) were selected for analysis. Oligo-tagged probes for each ROI were collected in 96-well plates and subjected to next-generation sequencing (NGS) preparation. Figure created with BioRender.

In other tumor subtypes, culture in HPLM has significant effects on cellular metabolism [6], and analysis using stable isotope labeled metabolites in HPLM can reveal tumor metabolic pathway activity [14]. Measuring the contribution of isotopically labeled nitrogen from glutamine to diverse metabolic pathways has emerged as a valuable tool to study cancer cell metabolism [15, 16]. Proliferating glioma cells use glutamine as a substrate for the synthesis of nucleosides, non-essential amino acids, and glutathione, among other pathways. Additionally, evidence has emerged that culture in HPLM also has immunomodulatory effects, including enhancing T cell activation following antigen stimulation through diverse transcriptional responses [17]. Here, we cultured SXOs in SXO-adapted HPLM to enable stable isotope tracing (Fig. 1). We found that SXOs cultured in HPLM maintain histologic characteristics and TME cell constituents, increase immune cell signaling, and display stable metabolic activity during prolonged HPLM culture.

## Methods

### Human Subjects.

Patient tissue and blood were collected following ethical and technical guidelines on the use of human samples for biomedical research at the University of Texas Southwestern Medical Center after informed patient consent under a protocol approved by the University of Pittsburgh’s Institutional Review Board (MOD19080321–003) and the University of Texas Southwestern Medical Center’s IRB (STU 022011–070). Supplementary Table 1 summarizes relevant clinical and demographic information for primary SXO and cell culture models developed at UT Southwestern Medical Center.

### Primary Cell Culture.

TS516 (sex unknown) cells (RRID:CVCL_A5HY) were obtained from I. Mellinghoff at Memorial Sloan-Kettering Cancer Center [18]. HK157 (female) cells were obtained from H. Kornblum at the University of California Los Angeles [19]. TS516, HK157, and UTSW63 cells were cultured in NeuroCult NS-A Basal Medium (Human) with Proliferation Supplement (StemCell Technologies 05751), supplemented with EGF (20 ng/mL, GoldBio 1150–04-100), bFGF (20 ng/mL, GoldBio 1140–02-10), heparin (2 mg/mL, STEMCELL Technologies 07980), 1% penicillin/streptomycin, amphotericin B (250 ng/mL, Gemini Bio-Products 400104), and Plasmocin (2.5 mg/mL, Invitrogen ant-mpt-1).

### SXO Creation and Culture in GOC.

Tumor tissue was collected from the operating room, directly suspended in ice cold Hibernate A (BrainBits HA), transferred to the laboratory on ice within 30 minutes of explantation. Tumor pieces were moved into RBC lysis buffer (ThermoFisher 00433357) and incubated at room temperature for 10 minutes with rocking. Tumor pieces were then washed with Hibernate A containing Glutamax (final concentration 2 mM, ThermoFisher 35050061), penicillin/streptomycin (final concentration 100 U/mL and 100 μg/mL, respectively, ThermoFisher 15140122), and Amphotericin B (final concentration 0.25 μg/mL, Gemini Bio-Products 400104). Tissues were cut using a 750 μm^2^ internal diameter needle (SAI Infusion Technologies B18–150) and plated, one SXO per well, in a 24-well ultra-low adherence plate (Corning 3473) in 1 mL Glioma Organoid Complete Medium (GOC) [8]. Stocks of GOC were used up to 1 week after preparation. Plates were rotated at 120 rpm in a humidified incubator at 37°C, 5% CO_2_, and 5% oxygen. GOC was refreshed in SXO cultures every 48 hours. All SXOs were cultured for a minimum of eight weeks prior to experimentation.

### Culture in Glioma Stem-Like Cell (GSC) HPLM:

Human Plasma-Like Medium (HPLM) was prepared as previously described [6, 20], without the addition of dialyzed fetal bovine serum and glutamate. To this basal HPLM, 1X B27, 0.25X N2, 1% penicillin/streptomycin, amphotericin B (250 ng/mL), Plasmocin (2.5 mg/mL), EGF (20 ng/mL), bFGF (20 ng/mL), and heparin (2 mg/mL) was supplemented. For ^15^N_2_ glutamine isotopically labeled GSC HPLM, GSC HPLM was prepared as above, substituting equimolar ^15^N_2_ glutamine (Cambridge Isotope Laboratories NLM-1328) for unlabeled glutamine. Stocks of GSC HPLM were used up to 1 week after preparation. Upon first culture in GSC HPLM, GSCs were acclimated for 48 hours, first with 24 hours of exposure to a mixture of 50% NeuroCult and 50% GSC HPLM, followed by 24 hours of exposure to 100% GSC HPLM.

### Culture in SXO HPLM.

Human Plasma-Like Medium (HPLM) was prepared as previously described [6, 20], without the addition of dialyzed fetal bovine serum. To 500mL of this basal HPLM, 10mL B-27 Supplement without Vitamin A (ThermoFisher 12587010) and 5mL N-2 Supplement (ThermoFisher 17502048) were added. To 48mL of this supplemented HPLM, 48μL of 2-mercaptoethanol (final concentration 55μM, ThermoFisher BP176–100), and 12μL human insulin (final concentration 2.375–2.875μg/mL, Sigma Aldrich I9278) were added to make SXO HPLM. For ^15^N_2_ glutamine isotopically labeled SXO HPLM, SXO HPLM was prepared as above, substituting equimolar ^15^N_2_ glutamine (Cambridge Isotope Laboratories NLM-1328) for unlabeled glutamine. Stocks of SXO HPLM were used up to 1 week after preparation. Upon first culture in SXO HPLM, SXOs were acclimated for 48 hours, first with 24 hours of exposure to a mixed media of 50% GOC and 50% SXO HPLM, followed by 24 hours of exposure to 100% SXO HPLM.

### Histology and Immunohistochemistry.

SXOs were fixed in 10% formalin for 1 hour, washed, and suspended in 70% ethanol. Samples were embedded in paraffin and sectioned at 4μm prior to staining. Embedding, sectioning, histology, immunohistochemistry, and digital image acquisition were performed by HistoWiz Inc. (histowiz.com). Images were processed using FIJI (1.53f51, imagej.net/software/fiji, RRID:SCR_002285). Nuclei and immunohistochemical stains were quantified with a semi-automated trained object classifier to identify nuclei and determine positivity for diaminobenzidine, implemented in QuPath (0.3.1, qupath.github.io, RRID:SCR_018257).

### Liquid Chromatography-Mass Spectrometry (LC-MS).

For metabolomic and stable isotope tracing experiments, SXOs and GSCs were snap-frozen and stored at −80°C until analysis. For preparation of SXOs, accurate masses of snap frozen SXOs were obtained using an analytical balance. 100μL 80% LC-MS grade acetonitrile (Fisher Scientific A9554) prepared in LC-MS grade water (Fisher Scientific W6500) per mg of tissue was added to snap frozen SXOs on ice, after which SXOs were homogenized by manual agitation. Homogenized samples were vortexed at 4°C for 20 minutes, then centrifuged for 10 minutes at 21,100×g at 4°C. The supernatant was transferred to a fresh microcentrifuge tube and centrifuged again for 10 minutes at 21,100×g at 4°C. For GSCs, neurospheres were harvested from 6-well plates, followed by the addition of 4°C saline to quench metabolic activity. Samples were transferred to microcentrifuge tubes and centrifuged for 1 minute at 21,100×g at 4°C. The resulting supernatant was aspirated, with the remaining cell pellets snap frozen in liquid nitrogen and stored at −80°C. Metabolites were extracted in 80% Acetonitrile at a concentration of 1,000 cells/μL, vortexed for 20 minutes at 4°C, and centrifuged (21,100×g for 10 minutes at 4°C). The supernatant was transferred to a fresh microcentrifuge tube and centrifuged again (21,100×g for 10 minutes at 4°C). The supernatant was transferred to a glass vial for LC-MS analysis. 10μL of SXO sample or 20uL of GSC sample was injected and analyzed with an Q Exactive^™^ HF-X orbitrap mass spectrometer (ThermoFisher) coupled to a Vanquish ultra-high performance liquid chromatography system (ThermoFisher). Peaks were integrated using El-Maven software (0.12.0, Elucidata). Total ion counts were quantified using TraceFinder software (5.1 SP2, ThermoFisher). Peaks were normalized to total ion counts using the R statistical programming language. Correction for the natural abundance of ^15^N isotopes was accomplished using the R script Accucor [21]. Total fractional enrichment was calculated by summing fractional enrichment of isotopically labeled nitrogen in each isotopologue of the metabolite.

### Digital Spatial Profiling (DSP) Assay.

RNA spatial expression profiles were analyzed using GeoMx DSP (NanoString Technologies, Seattle, WA, USA, RRID:SCR_021660). After conventional deparaffinization and rehydration, 4-μm formalin-fixed paraffin-embedded (FFPE) tissue slides were hybridized and incubated at 37°C overnight with morphological immunofluorescent biomarkers and DSP probes from the Cancer Transcriptome Atlas panel (NanoString Technologies, Seattle, WA). Immunofluorescence biomarkers including the DNA marker SYTO13, the tumor marker GFAP, and the immune cell marker CD45 were used as visualization markers. Slides were then loaded onto the DSP instrument and scanned to produce a digital image displaying tissue with histological features highlighted by fluorescent visualization markers. Spatially resolved ROIs were selected on the slides based on fluorescent markers and consecutive H&E-stained slides. DSP probes conjugated to target-specific activated oligos were collected for each ROI and aliquoted into 96-well plates. Collection plates were dehydrated at 65°C for 1–2 hours on a thermal cycler with an open top and a breathable AeraSeal film (Excel Scientific, A9224) applied. Samples were reconstituted with diethyl pyrocarbonate-treated RNase/DNase-free H_2_O and library preparation was completed with eighteen cycles of amplification. Libraries were quantified on an Agilent 4200 TapeStation and pooled for sequencing on an Illumina NextSeq 2000 with a P3 50 flow cell. Whole transcriptome gene sequencing was performed on individual ROI tubes by the University of Pittsburgh Health Sciences Sequencing Core, Rangos Research Center, UPMC Children’s Hospital of Pittsburgh, Pittsburgh, Pennsylvania, United States of America. The resulting FASTQ files were decoded and processed into count files using the NanoString GeoMx NGS Pipeline (2.0.21) in the Illumina BaseSpace Sequencing Hub. Count files were uploaded to the GeoMx DSP instrument and indexed to corresponding slide scans for analysis. Obtained gene counts were mapped to selected ROIs and normalized after a quality check for the following parameters in each ROI: raw read count > 1,000, >80% of reads aligned, sequencing saturation >50%, negative probe geometric mean >10 in the background, count of nuclei per ROI >200, and surface area per ROI <16,000 μm^2^. The cell population, immune signatures, differentially expressed genes, and corresponding enriched pathways were determined in each selected ROI. CIBERSORT (RRID:SCR_016955), a deconvolution algorithm built on nine normalized gene expression profiles to characterize cell compositions [22], was used to estimate cell population proportions based on the leukocyte signature matrix 22 (LM22). CIBERSORT was run for 1,000 permutations, and samples with a CIBERSORT *p*-value below 0.05 were included for subsequent analyses. Gene expression analysis was performed using the GeoMx Analysis suite (2.4.2.2 , RRID:SCR_023424). Gene set enrichment analysis (GSEA, 4.2.3, Broad Institute, Inc., Massachusetts Institute of Technology, and Regents of the University of California, RRID:SCR_003199) based on the Molecular Signatures Database (RRID:SCR_016863) was performed to compare SXOs cultured in HPLM to SXOs cultured in GOC.

### Quantification and Statistical Analysis.

SXOs were allocated to experiments randomly and samples were processed in an arbitrary order. All statistical tests were two-sided, where applicable. Student’s *t*-test was used to assess the statistical significance of a difference between the two groups. Linear regression analysis and Pearson’s correlation coefficient were used to assess correlation between the two groups. Statistical analyses were performed with GraphPad Prism (9.2.0.332, GraphPad Software, LLC) and included both descriptive statistics as well as tests of statistical significance. All data are plotted as mean ± standard deviation. For all tests, *p-*values less than 0.05 were considered statistically significant.

## Results

We established a SXO model, SXO210, from a 68-year-old male patient diagnosed with a right temporal glioblastoma, IDH wild-type, grade 4 (Supplementary Table 1). Clinical pathology was consistent with glioblastoma, highlighted by GFAP immunostaining, a high mitotic index, areas of pseudopalisading necrosis, geographic necrosis, and microvascular proliferation. From this tumor, we successfully generated SXOs, which were cultured in GOC medium for 8 weeks prior to initiating these studies.

### SXO cellular architecture is preserved after culture in SXO HPLM

**Fig. 2:**
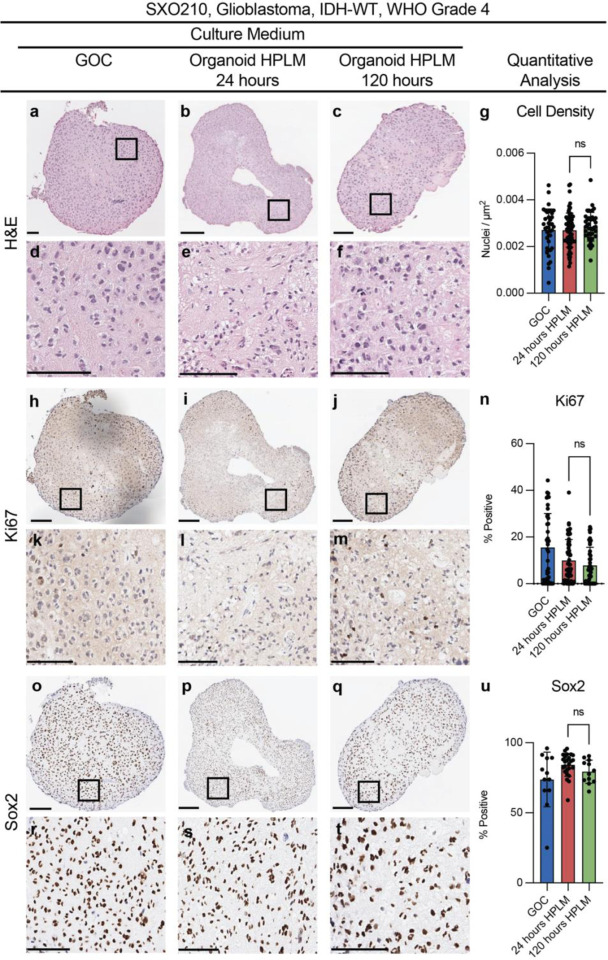
SXO histology after culture in SXO HPLM SXOs cultured in GOC with glutamate or SXO HPLM for 24 or 120 hours were fixed and stained for H&E, Ki67, and Sox2. **(a-f)** H&E stain of a representative SXO cultured in GOC **(a)**, HPLM for 24 hours **(b)**, and HPLM for 120 hours **(c)**, which are shown at high magnification in **(d-f)**. **(g)** Imaging-based quantification of cell density by evaluation of nuclei/μm^2^. **(j-m)** Ki67 immunohistochemistry (IHC) stain of a representative SXO cultured in GOC **(j)**, HPLM for 24 hours **(i)**, and HPLM for 120 hours **(j)**, which are shown at high magnification in **(k-m)**. **(n)** Imaging-based quantification of Ki67 through evaluation of the percentage of positive cells. **(r-t)** Sox2 IHC stain of a representative SXO cultured in GOC **(r)**, HPLM for 24 hours **(p)**, and HPLM for 120 hours **(q)**, which are shown at high magnification in **(r-t)**. **(u)** Imaging-based quantification of Sox2 through evaluation of the percentage of positive nuclei. Cell density, Ki67 positivity, and Sox2 positivity were quantified by a semi-automated trained object classifier. Data are presented as means ± standard deviation, ns: not significant; n = 3; two-tailed *p*-values were determined by unpaired *t*-test. Scale bar = 100μm.

We assessed SXO architecture and viability after culture in SXO HPLM, compared to in GOC, by performing histological analyses, which were reviewed by a board-certified neuropathologist. Each SXO was independently stained with hematoxylin and eosin (H&E). SXOs cultured in SXO HPLM maintained typical histologic features of glioblastoma, including necrosis, microvascular proliferation, high cellularity with abundant mitosis, and nuclear atypia (Fig. 2e–f). Quantitative analysis of cellular nuclei demonstrated that SXOs cultured in SXO HPLM for either 24 or 120 hours maintained similar cell density to those cultured in GOC, indicating limited impact of short- and medium-term HPLM culture on cellularity (Fig. 2g). SXOs were subsequently stained for cellular proliferation using Ki67 (Fig. 2h–m). Quantitative bioimage analysis of the proportion of cells with positive staining for Ki67 between the GOC and both SXO HPLM-cultured groups were not significantly different, indicating a similar degree of cell proliferation upon short- and medium-term exposure to HPLM (Fig. 2n). Finally, SXOs were stained for the marker of the stem-like cell fraction, Sox2 (Fig. 2o–2t). The percentage of cells positive for Sox2 staining was equivalent between SXOs cultured in GOC and in SXO HPLM, suggesting that the stem-like cell population was not significantly altered upon culture in HPLM (Fig. 2u).

### SXOs cultured in HPLM maintain tumor microenvironment cellular composition and upregulate immune transcriptional programs

**Fig. 3:**
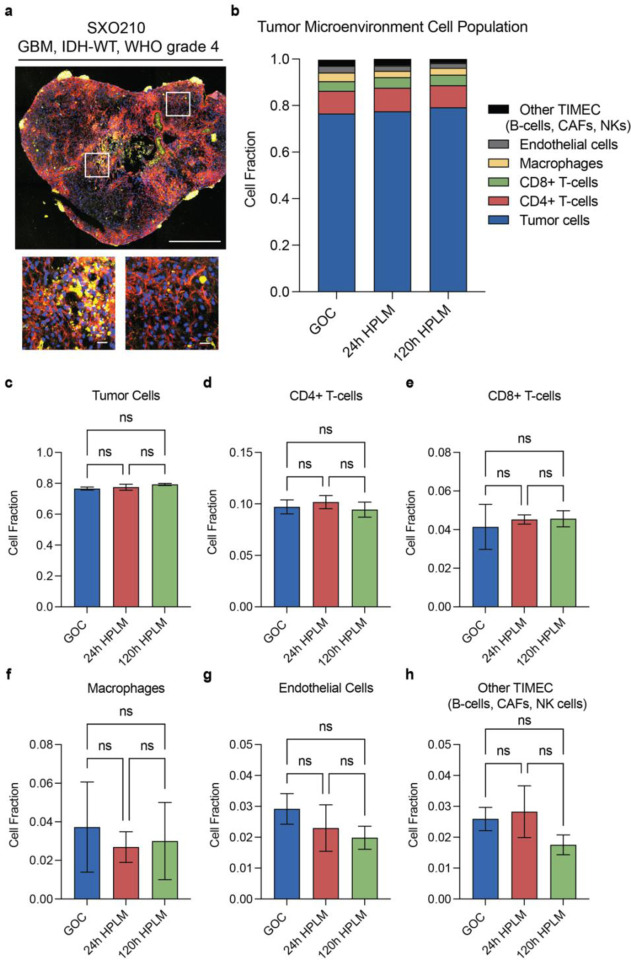
SXO cell populations after culture in HPLM **(a)** Representative morphology scan of cell populations in SXO210 cultured in HPLM on the GeoMx Digital Spatial Profiler. Red: GFAP (tumor cells), Yellow: CD45 (immune cells), Blue: CYTO-13 (nuclear stain). **(b-h)** Transcriptional signature analysis to assign identities to TME cell populations in SXOs cultured in GOC or HPLM. **(b)** Distribution of all cell identities. Fraction of tumor cells **(c)**, CD4+ T-cells **(d)**, CD8+ T-cells **(e)**, macrophages **(f)**, endothelial cells **(g)**, and other tumor immune microenvironment cells [TIMEC, including B-cells, cancer-associated fibroblasts (CAFs), and natural killer (NK) cells] **(h)**. Data are presented as mean ± standard deviation, ns: not significant; n = 3; *p* value was determined by one-way ANOVA. Scale bar = 250μm; insets = 50μm.

**Fig. 4:**
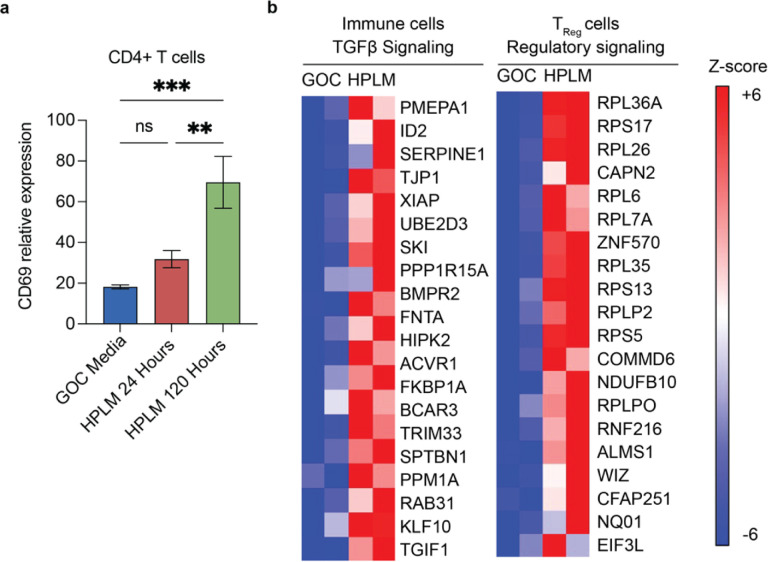
Immune signaling in SXOs after culture in HPLM **(a)** Relative expression of the early activation marker CD69 in cells classified as CD4+ T cells **(b)** Normalized Enrichment Score (NES) heatmap of genes related to TGF-β signaling in all immune cells captured and T regulatory (T_reg_) cell signaling within T_reg_ cells in SXOs cultured in GOC and HPLM for 120 hours.

To assess the TME of SXOs cultured in SXO HPLM or GOC, we performed spatial transcriptomics with a NanoString GeoMx digital spatial profiler (DSP). We used immunofluorescent probes for the marker SYTO13 to identify all cells, GFAP to specifically mark tumor cells [23], and CD45 to specifically identify immune cells [24] on fixed SXO sections cultured in either GOC, HPLM for 24 hours, or HPLM for 120 hours (Fig. 3a). We then scanned these regions to construct digital maps of cellular content and selected regions of interest (ROIs) containing diverse heterocellular populations representative of the whole SXO or regions exclusively containing immune cells. Individual ROIs were collected for independent transcriptional analysis by next-generation sequencing. Using next-generation sequencing of representative heterocellular SXO ROIs, we classified cell types present using the CIBERSORT digital cytometry platform [25]. We found that SXOs contained a diverse, spatially variable population of constituent cells, consistent with prior work [10, 26]. 20–30% of cells in each SXO were identified as non-tumor, including CD4+ T-cells, CD8+ T-cells, and macrophages/microglia (Fig. 3b). SXOs cultured in SXO HPLM and GOC maintained similar proportions of tumor cells (Fig. 3c), CD4+ T-cells (Fig. 3d), CD8+ T-cells (Fig. 3e), macrophages/microglia (Fig. 3f), endothelial cells (Fig. 3g), and other tumor immune microenvironment cells (TIMEC), including B-cells, cancer-associated fibroblasts (CAFs), and natural killer (NK) cells (Fig. 3h). Activation of CD4+ T-cells can be marked by increased expression of CD69 [27]. We observed increased expression of CD69 in CD4+ T cells of SXOs cultured in HPLM for 120 hours, but not 24 hours (Fig. 4a). TGF-β signaling in bulk immune cell populations and T regulatory (T_reg_) cell signaling in T_reg_ cells were significantly enriched in SXOs cultured in HPLM for 120 hours relative to SXOs cultured in GOC (Fig. 4b). Gene set enrichment analysis revealed increased expression of pathways related to the innate immune system, adaptive immune system, and cytokine signaling in these cells (Table 1). These data indicate that culture of SXOs in HPLM retains TME heterogeneity while inducing immunologic transcriptional programs.

### Measurement of isotopic labeling by liquid chromatography-mass spectrometry (LC-MS) in SXOs cultured in HPLM

**Fig. 5:**
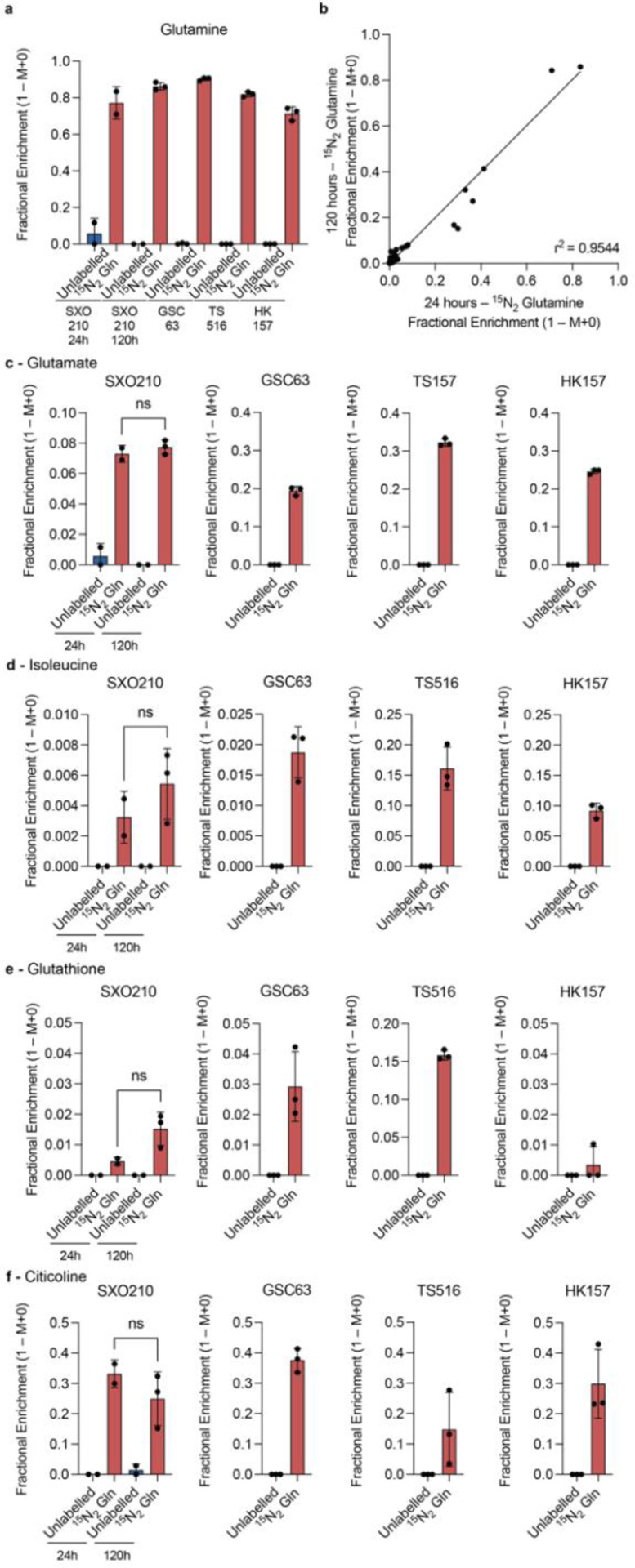
SXO and GSC stable isotope tracing in HPLM LC-MS analysis of SXOs or GSCs acclimated to HPLM, cultured for either 24 or 120 hours in HPLM, then cultured for 24 hours in ^15^N_2_ glutamine isotopically labeled HPLM. **(a)** Fractional enrichment of ^15^N isotopic labeling by glutamine in all isotopologues of glutamine. **(b)** Linear regression analysis comparing fractional enrichment of total ^15^N isotopic labeling in all nitrogen-containing metabolites detected between SXOs cultured for 24 or 120 hours in SXO HPLM. **(c-f)** Fractional enrichment of ^15^N isotopic labeling after 24 hours of stable isotope tracer exposure in SXOs or GSCs in the sum of all isotopologues of glutamate **(c)**, isoleucine **(d)**, glutathione **(e)**, and citicoline **(f)**. *r*: Pearson’s correlation coefficient; data are presented as means ± standard deviation; ns: not significant, n = 2 for unlabeled groups and 24-hour SXO HPLM group, n = 3 for 120-hour SXO HPLM group and all GSCs; two-tailed *p*-values were determined by unpaired *t*-test.

We utilized LC-MS to quantify metabolite levels and paired ^15^N isotopically labeled metabolite levels for a diverse set of metabolites in SXOs cultured in HPLM, utilizing three GSC lines (UTSW63, TS516, and HK157) as controls for label accumulation. We selected metabolites expected to be enriched for isotopic labeling from glutamine for further analysis. We first confirmed intra-organoid and intracellular accumulation of the isotopic label in glutamine, which was present in culture conditions containing the ^15^N_2_ glutamine stable isotope tracer and not in unlabeled conditions (Fig. 5a). We then determined the fractional enrichment of isotopic label derived from ^15^N_2_ glutamine in the non-essential amino acids alanine (Supplementary Fig. 1a), asparagine (Supplementary Fig. 1b), and glutamate (Fig. 5c), the branched-chain amino acid isoleucine (Fig. 5d), the redox substrate glutathione (Fig. 5e), the nucleoside citicoline (Fig. 5f), the glutamate derivative N-acetylglutamate (Supplementary Fig. 1c). We compared nitrogen-labeled isotopologues of these metabolites in SXOs or GSCs cultured in HPLM with ^15^N_2_ glutamine stable isotope tracer to those of SXOs or GSCs cultured in HPLM without tracer. Despite adequate detection of the parental metabolite, there was no significant labeling observed in the no tracer condition (Figs. 5c–f, Supplementary Figs. 1a–c). All SXOs cultured with stable isotope tracer exhibited significant fractional enrichment of one or more isotopologues, mirroring patterns of label accumulation evident in GSC lines (Fig. 5c–f, Supplementary Figs. 1a–c).

### Metabolic activities are similar between SXOs cultured for 24 and 120 hours in SXO HPLM

To evaluate whether SXOs preserve metabolic characteristics between short- and medium-term culture in HPLM, we compared label accumulation after 24 hours of stable isotope tracer exposure between SXOs preconditioned for either 24 or 120 hours in SXO HPLM. Linear regression analysis comparing all detected nitrogen-containing metabolites by LC-MS demonstrated no significant difference in the fractional enrichment of total ^15^N isotopic labeling between SXOs cultured for 24 or 120 hours in SXO HPLM, with an *r*^2^ of 0.9544 (Fig. 5b). In the metabolites representing a diverse array of biosynthetic pathways including nonessential amino acid biosynthesis, branched-chain amino acid transamination, redox homeostasis, nucleotide synthesis, and the urea cycle, there was no significant difference in the fractional enrichment of ^15^N isotopic labeling between SXOs cultured for either 24 or 120 hours in SXO HPLM (Fig. 5c–f, Supplementary Figs. 1a–c). These data indicate that HPLM can sustain SXO metabolism during prolonged culture, enabling stable isotope tracing studies to be performed under steady-state, homeostatic conditions.

## Discussion

Biochemical networks have drawn interest as sources of new therapeutic targets in glioma. Much progress has been made in understanding tumor metabolism using stable isotope tracing in cell culture and in vivo mouse models. Cell culture models do not capture the diverse heterocellular environment of parental tumors and often are performed in media conditions that favor rapid cell growth and division and do not mirror physiologic extracellular nutrient availability. Orthotopic xenograft murine models are expensive and technically challenging to utilize, particularly for stable isotope tracing studies, and rely on immunocompromised host mice, limiting the evaluation of tumor-immune interactions in the TME. Advanced model systems that more closely mirror the physiologic environment of brain tumors may contribute to our understanding of metabolic pathways relevant to tumorigenesis and tumor cell fitness [15, 28]. Here we demonstrate patient-derived SXOs and GSCs can be transitioned to HPLM, a culture medium designed to mirror physiologic extracellular nutrient availability. We show that transitioning these SXOs to HPLM does not compromise characteristics that render them attractive for the study of tumor biology: maintenance of local cytoarchitecture, cellular heterogeneity, cell-cell, and cell-stroma interactions, and TME constituent cells. Further, we demonstrate that medium-term culture in HPLM increases immune transcriptional programs within SXO immune cells, potentially rendering these models more attractive for the study of tumor-immune interactions and immunometabolism. Finally, we performed stable isotope tracing studies in SXOs and GSCs cultured in HPLM, demonstrating durable label accumulation in diverse metabolic pathways.

Analysis of metabolism in SXOs may provide insights into how gliomas alter typical neural cell metabolic programs to foster growth and proliferation. This approach also captures features of whole tumor metabolism driven by microenvironmental components that are lacking in pure tumor cell cultures. Evaluating the biochemical networks active in these primary models under physiological nutrient levels may aid in the identification of novel tumor-specific metabolic phenotypes and associated targetable vulnerabilities.

## Limitations

We acknowledge that our study comes with limitations. The number of SXOs utilized was limited (n = 9 for both histology and stable isotope tracing), and all experimental replicates derive from a single patient, thus potentially underpowering this study to detect true differences in organoid viability and metabolic activity. We evaluated culture of SXOs and GSCs in HPLM for a maximum of 120 hours. This limited length may fail to identify the impact of longer-term adaptation on SXO metabolic activity or viability.

## Conclusion

We demonstrated ex vivo stable isotope tracing under conditions that recapitulate the in vivo TME using SXOs grown in HPLM. The provision of nutrients at levels that mimic in vivo settings triggered an activation-related transcriptional response in immune cells present in SXOs. Our work offers an approach for evaluating whole tumor metabolism that addresses key challenges associated with conventional methods.

## Supplementary Material

Supplement 1

Supplement 2

## Figures and Tables

**Table 1.** Gene set enrichment analysis of transcripts in SXOs cultured in HPLM for 120 hours versus GOC, displaying the five most significantly regulated immune pathways per category.

Reactome Pathway ID Description	NES	PC (%)	Adjusted *p*-value
**Innate Immune System**			
Fc epsilon receptor (FCERI) signaling	2.78	98.5	0.0008
FCERI mediated NF-kB activation	2.74	97.6	0.0008
MAP kinase activation	2.56	96.9	0.0008
Toll Like Receptor 10 (TLR10) Cascade	2.31	95.4	0.0016
Toll Like Receptor 5 (TLR5) Cascade	2.31	95.4	0.0016
**Adaptive Immune System**			
Antigen processing: Ubiquitination & Proteasome degradation	4.01	98.4	0.0008
Class I MHC mediated antigen processing & presentation	3.22	98.1	0.0008
Downstream signaling events of B Cell Receptor (BCR)	3.14	98.8	0.0008
Activation of NF-kB in B cells	3.10	98.5	0.0008
Signaling by the B Cell Receptor (BCR)	2.35	99.1	0.0008
**Cytokine Signaling**			
NIK-->noncanonical NF-kB signaling	3.29	98.3	0.0008
Interleukin-1 signaling	2.66	97.1	0.0008
Interleukin-17 signaling	2.22	97.2	0.0021
Interferon alpha/beta signaling	2.01	92.9	0.0070
Interleukin-1 family signaling	1.96	97.9	0.0066

## Data Availability

The histopathology images will be shared by the lead contact upon request. Any additional information required to reanalyze the data reported in this paper is available from the lead contact upon request.
