# Supplementary material for: Human plasma-like medium facilitates metabolic tracing and enables upregulation of immune signaling pathways in glioblastoma explants
Source: bioRxiv. 2023 May 31:2023.05.29.542774. Preprint. [Version 1] doi: 10.1101/2023.05.29.542774 (PMC10312566; doi:10.1101/2023.05.29.542774)
Supplement: Supplementary file 1 [file media-1.pdf]

Supplementary Figure 1

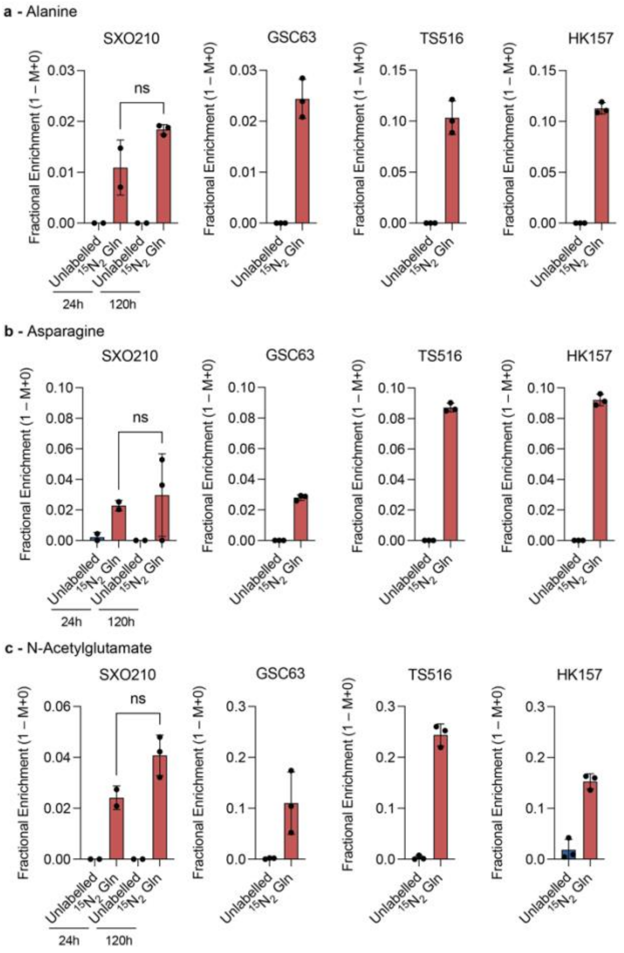

Supplementary Fig. 1 SXO and GSC stable isotope tracing in HPLM LC-MS analysis of SXOs or GSCs acclimated to HPLM, cultured for either 24 or 120 hours in HPLM, then cultured for 24 hours in $^{15}N_2$ glutamine isotopically labeled HPLM. **(a-c)** Fractional enrichment of ^{15}N isotopic labeling after 24 hours of stable isotope tracer exposure in SXOs or GSCs in the sum of all isotopologues of alanine **(a)**, asparagine **(b)**, and N-acetylglutamate **(c)**. Data are presented as means ± standard deviation; ns: not significant, n = 2 for unlabeled groups and 24-hour SXO HPLM group, n = 3 for 120-hour SXO HPLM group, and all GSCs; two-tailed *p*-values were determined by unpaired *t*-test.
