# Supplementary material for: Human plasma-like medium facilitates metabolic tracing and enables upregulation of immune signaling pathways in glioblastoma explants
Source: bioRxiv. 2023 May 31:2023.05.29.542774. Preprint. [Version 1] doi: 10.1101/2023.05.29.542774 (PMC10312566; doi:10.1101/2023.05.29.542774)
Supplement: Supplementary file 2 [file media-2.pdf]

Supplementary Table 1

Sample ID	Model	Age	Sex	Brain Region	WHO Grade	Diagnosis	Primary/ Recurrent
GSC63	Cell Line	71	M	RT	4	GBM, IDH-wildtype	Primary
SXO210	SXO	68	M	RT	4	GBM, IDH-wildtype	Primary

Supplementary Table 1. Clinical characteristics of primary tumor specimens used for SXO and GSC lines derived in this study. “Brain Region” corresponds to the location of the tumor at resection (RT, right temporal). “Diagnosis” corresponds to the results of histopathologic features and molecular studies rendered by a clinical pathologist at resection. Abbreviations: M, male; GBM, glioblastoma; IDH, isocitrate dehydrogenase. IDH status of each primary tumor sample was assessed via IHC, next-generation DNA sequencing, or both.
